# Cell–cell interactions between monocytes/macrophages and synoviocyte-like cells promote inflammatory cell infiltration mediated by augmentation of MCP-1 production in temporomandibular joint

**DOI:** 10.1042/BSR20171217

**Published:** 2018-03-29

**Authors:** Miho Ibi, Sawa Horie, Seiko Kyakumoto, Naoyuki Chosa, Mariko Yoshida, Masaharu Kamo, Masato Ohtsuka, Akira Ishisaki

**Affiliations:** 1 Division of Cellular Biosignal Sciences, Department of Biochemistry, Iwate Medical University, 2-1-1 Nishitokuta, Yahaba-cho, Shiwa-gun, Iwate 028-3694, Japan; 2 Department of Tumor Biology, Institute of Biomedical Science, Iwate Medical University, 2-1-1 Nishitokuta, Yahaba-cho, Shiwa-gun, Iwate 028-3694, Japan; 3 Division of Cellular Physiology, Department of Physiology, Iwate Medical University, 2-1-1 Nishitokuta, Yahaba-cho, Shiwa-gun, Iwate 028-3694, Japan; 4 Department of Molecular Life Science, Division of Basic Medical Science and Molecular Medicine, School of Medicine, Tokai University, 143 Shimokasuya, Isehara, Kanagawa 259-1193, Japan; 5 The Institute of Medical Sciences, Tokai University, 143 Shimokasuya, Isehara, Kanagawa 259-1193, Japan

**Keywords:** monocyte/macrophage, MCP-1, synoviocyte-like cells, temporomandibular joint

## Abstract

Many inflammatory cells are known to be home to inflamed temporomandibular joint (TMJ) tissues by stimulation with cytokines and chemokines produced by inflammatory lesions in the TMJ. However, how the inflammatory cells affect the progression of inflammation in TMJ synovial tissues after their homing to inflamed TMJ site is still uncertain. Here, we isolated and cultured TMJ synoviocyte-like cells (TMJSCs) from murine TMJ tissues. We demonstrated that interleukin 1β (IL-1β) up-regulated expression of monocyte chemoattractant protein 1 (MCP-1) in TMJSCs. In addition, we found that IL-1β-treated TMJSCs strongly promoted migratory activity of mouse monocyte/macrophage RAW264.7 cells through secretion of MCP-1. On the other hand, IL-1β up-regulated expression levels of intracellular adhesion molecule 1 (ICAM-1), a leukocyte adhesion ligand in TMJSCs. In addition, IL-1β promoted cell–cell adhesion between TMJSCs and RAW264.7 cells. Intriguingly, we also found that cell–cell interactions mediated through soluble factors other than IL-1β and cell–cell adhesion molecules between IL-1β-stimulated TMJSCs and RAW264.7 cells synergistically augmented secretion of MCP-1 from these cells. Therefore, these results suggested that the IL-1β-induced recruitment of monocyte/macrophage lineage cells to inflamed synovial membranes in TMJ was further augmented by the cell–cell interaction-induced secretion of MCP-1 from the inflammation site, possibly resulting in prolonged inflammatory responses in TMJ synovial tissue.

## Introduction

The temporomandibular joint (TMJ) is a synovial joint that is constituted between the mandibular fossa of the temporal bone and mandibular condyle. The intra-articular structures of TMJ are covered by the synovial membrane except for the articular cartilage of the eminence and fossa, the mandibular condyle, and the articular disc [1,2]. Temporomandibular disorders (TMD) are prevalent oral diseases, and the discomfort of TMJ pain, joint noise, and limitation to opening the mouth affect daily living, including eating and speaking. Arthroscopic or histopathological findings in patients of internal derangement or osteoarthritis (OA) of TMJ revealed synovitis, i.e. inflammation of the synovial membrane [3,4]. Furthermore, many proinflammatory cytokines have been detected in the synovial fluid of patients with TMD [5].

Recently, it has been reported, through microarray analysis, that mRNAs of cell migration-inducible chemokines, including monocyte chemoattractant protein 1 (MCP-1, also known as CCL2), are highly expressed in human TMJ synoviocytes stimulated with interleukin 1β (IL-1β) or tumor necrosis factor-α (TNF-α) [6–8]. Previous studies have also shown that inflammatory cells such as lymphocytes and macrophages infiltrate into the synovial lining in TMJ patients [4,9]. Therefore, it is expected that there is a certain interaction between TMJ synoviocytes and inflammatory cells that are home to the TMJ inflammation site for augmentation of local inflammation. However, the inflammatory molecular mechanisms underlying interactions between the TMJ synovial cells and chemokine-homed immune cells remain poorly understood.

To elucidate how the infiltrated inflammatory cells affect the condition of synovitis or its transition to chronic inflammation in TMJ, we investigated the inflammatory interaction between IL-1β-stimulated TMJ synoviocyte-like cells (TMJSCs) obtained from mouse TMJ and the murine macrophage cell line RAW264.7. Here, we report that IL-1β stimulation of TMJSCs facilitated the adhesion of RAW264.7 cells on to TMJSCs. Furthermore, the direct contact between IL-1β-stimulated TMJSCs and RAW264.7 cells promoted further production of MCP-1, which possibly induced further infiltration of inflammatory cells to the inflamed synovial membrane in TMJ, resulting in chronic inflammation.

## Materials and methods

### Mice

C57BL/6J EGFP transgenic mice were generated by a recombinase-mediated cassette exchange (RMCE) method [10]. Ten-week-old EGFP male mice was used in the present study. Nine-week-old C57BL/6J female mice was purchased from Nihon Crea Co. (Tokyo). EGFP and C57BL/6J mice were maintained and studied at Iwate Medical University under the guidelines and approved protocols of the Committee on Animal Experiments of Iwate Medical University, Morioka, Japan.

### Antibodies, cytokines, chemokines

Rat anti-mouse CD54 (intracellular adhesion molecule 1 (ICAM-1)) antibody (clone #: YN1/1.7.4, rat IgG_2b, κ_) was purchased from BioLegend (San Diego, CA, U.S.A.). Rat anti-mouse CD45 (clone #: 30-F11) was obtained from BD Biosciences Pharmingen (San Diego, CA, U.S.A.). Rabbit anti-GFP (ab6556) was purchased from Abcam (Cambridge, MA, U.K.). Alexa Fluor® 594 Donkey anti-Rat IgG, Alexa Fluor®488 Donkey anti-Rabbit IgG and Alexa Fluor®488 phalloidin were sourced from Molecular Probes (Invitrogen, U.S.A.). Recombinant mouse IL-1β and MCP-1 were purchased from PROSPEC (East Brunswick, NJ, U.S.A.). Recombinant human fibroblast growth factor 2 (rhFGF-2) was obtained from Miltenyi Biotec (Bergisch-Gladbach, Germany).

### Cell preparation and culture

After EGFP mice were sacrificed under anesthesia, the tissue surrounding the TMJ was obtained and washed extensively with PBS (Nissui Pharmaceutical Co., Ltd., Tokyo, Japan), and then immersed in digestion solution comprising 0.21% collagenase (Wako Pure Chemical, Osaka, Japan) in Dulbecco’s modified Eagle’s medium (DMEM, Sigma Chemicals, St. Louis, MO, U.S.A.) for 40 min at 37°C with continuous vigorous rocking. Then, the digested explants were cultured in DMEM supplemented with 20% FBS (PAA Laboratories Inc., Ontario, Canada) and antibiotics (Gibco, Carlsbad, CA, U.S.A.) in a humidified atmosphere of 5% CO_2_ at 37°C. The cells that had grown out from the digested explants and reached subconfluence were detached from the surface of plastic tissue culture plates with 0.25% trypsin and 0.02% EDTA (Gibco, Carlsbad, CA, U.S.A.). We termed these cells as TMJSCs. TMJSCs were subcultured with 20% FBS in DMEM supplemented with rhFGF-2 (10 ng/ml) and antibiotics in a humidified atmosphere of 5% CO_2_ at 37°C. We observed cell morphology and obtained photomicrographs using an Olympus IX70 fluorescence microscope equipped with a DP72 digital camera (Olympus Corp., Tokyo, Japan). The cells from passages six to fifteen were used in the subsequent experiments.

Mouse embryonic fibroblast NIH3T3 cells and murine monocyte/macrophage cell line RAW264.7 cells were maintained in DMEM containing 10% FBS with antibiotics. For RNA isolation, these cells were grown to 80% confluence.

Total splenocytes were collected using 25G needles after spleen was harvested from C57BL/6J mouse. The splenocytes were filtered through a 70-μm mesh to remove any connective tissue or large aggregates. Contaminating red blood cells were removed using BD Pharm Lyse Lysing Buffer (BD Biosciences, San Jose, CA, U.S.A.) according to the manufacturer’s instructions. Briefly, the cells obtained from C57BL/6J mouse spleen were treated with BD Pharm Lyse Lysing Buffer for 3 min at 37°C. Thereafter, the cells were washed in PBS, splenocytes were collected for RNA isolation.

Resident peritoneal macrophages were isolated from the peritoneal cells obtained by flushing the peritoneal cavity of 12-week-old female C57BL/6J mice with cold PBS (5 ml per mouse). At first, we collected peritoneal cells from mouse peritoneal cavity. The peritoneal cells were centrifuged for 10 min at 4°C, then seeded into culture dish and preincubated in a humidified atmosphere of 5% CO_2_ at 37°C for 2 h with RPMI 1640 (Sigma Chemicals, St. Louis, MO, U.S.A.). After preincubation, the non-adherent cells were washed in RPMI 1640 medium or PBS several times. We used the adherent cells as resident peritoneal macrophages.

### RNA isolation and quantitative reverse transcriptase-PCR

Total RNA was isolated from cultured TMJSCs, NIH3T3 cells, RAW264.7 cells, and C57BL/6J mouse splenocytes, and resident peritoneal macrophages using ISOGEN II reagent (Nippon Gene, Toyama, Japan) according to the manufacturer’s instructions. The cDNA was prepared using the PrimeScript RT Reagent Kit (Takara-Bio, Shiga, Japan). PCR was performed on a Thermal Cycler Dice Real Time System (Takara-Bio, Shiga, Japan), using SYBR Premix Ex Taq II™ (Takara-Bio, Shiga, Japan) with specific oligonucleotide primers (mouse α1 chain of collagen type I (colIα1), 5′-GCTCCTCTTAGGGGCCACT-3′ (forward) and 5′-CCACGTCTCACCATTGGGG-3′ (reverse); mouse vimentin, 5′-ACCGCTTTGCCAACTACAT-3′ (forward) and 5′-TTGTCCCGCTCCACCTC-3′ (reverse); mouse CD45, 5′-GAACATGCTGCCAATGGTTCT-3′ (forward) and 5′-TGTCCCACATGACTCCTTTCC-3′ (reverse); mouse MCP-1, 5′-TTAAAAAACCTGGATCGGAACCAA-3′ (forward) and 5′-GCATTAGCTTCAGATTTACGGGT-3′ (reverse); mouse CC chemokine receptor 2 (CCR2), 5′- AGAGGTCTCGGTTGGGTTGT-3′ (forward) and 5′-CACTGTCTTTGAGGCTTGTTGC-3′ (reverse); mouse ICAM-1, 5′-GACAGCAGTCCGCTGTGCTT-3′ (forward) and 5′-GAGGTCTCAGCTCCACACTC-3′ (reverse); mouse GAPDH, 5′-TGTGTCCGTCGTGGATCTG-3′ (forward) and 5′-TTGCTGTTGAAGTCGCAGGAG-3′ (reverse); and mouse 18S rRNA, 5′-CGCCGCTAGAGGTGAAATTCT-3′ (forward) and 5′-CATTCTTGGCAAATGCTTTCG-3′ (reverse)). The mRNA expression levels of *MCP-1* and *ICAM-1* were normalized to that of *GAPDH* (Figures 2A and 3C), and those of other genes were normalized to 18S rRNA levels.

### IL-1β stimulation of TMJSCs

TMJSCs were seeded in 24-well plates (Nunc; Thermo Fisher Scientific) at a density of 5.3 × 10^3^ cells/well in 0.5-ml DMEM containing 20% FBS and rhFGF-2 (10 ng/ml) for 3 days. Then, after the cells were serum-starved (cultured in DMEM only) and incubated overnight (12–17 h), they were stimulated with a range of concentrations of IL-1β for 4 h (0.01–1 ng/ml; Figures 2A and 3C), 24 h (0.1 ng/ml; Figures 3A,B,D and 4), or 27 h (0.01 ng/ml; Figure 2B,E).

### Cell migration assay

Cell migration assays were performed using Transwell® membrane cell culture inserts (8 µm pore size; Corning, NY, U.S.A.) according to the manufacturer’s instructions. Briefly, TMJSCs were incubated in individual wells of a 24-well cell culture insert companion plate (Corning, NY, U.S.A.) in the same way as described in the ‘IL-1β stimulation of TMJSCs’ section, and stimulated with 0.01 ng/ml IL-1β for 24–30 h. Before migration assay, TMJSCs stimulated with 0.01 ng/ml IL-1β were washed with DMEM once. With regard to cell migration in response to recombinant mouse MCP-1 (rmMCP-1), DMEM containing 2% FBS and rmMCP-1 (100 ng/ml) was added to individual wells of a 24-well cell culture insert companion plate. Some RAW264.7 cells were preincubated with 1 µM RS504393, a selective CCR2 chemokine receptor antagonist (Sigma–Aldrich, St. Louis, MO, U.S.A.) for 1 h before the experiment (Figure 2E). Then, RAW264.7 cells were layered on the top of the membrane at 1 × 10^5^ cells/300 µl/well with serum-free DMEM and the transwell devices were inserted into the individual wells of the plates. Cells were allowed to migrate for 24 h into the lower surface of the filters. After migration, RAW264.7 cells that had mobilized to the lower surface of the filters were fixed in 3.7% formaldehyde (from 37% formaldehyde; Merck, Darmstadt, Germany) in PBS for 2 min and in methanol (Wako Pure Chemical, Osaka, Japan) for 20 min, and stained with Giemsa’s stain solution (Nacalai Tesque Inc., Kyoto, Japan) for 24 h at room temperature. RAW264.7 cells that had not migrated into the lower compartment were removed from the upper surface of the filters, using cotton swabs. Then, the cells that had migrated into the lower surface of the filters were observed and photographed using an OLYMPUS IX70 inverted fluorescence microscope equipped with a DP72 digital camera. The cells stained with Giemsa’s stain solution were counted as the number of migrated cells visualized on the photomicrographs.

### Quantitation of MCP-1 and IL-1β by ELISA

To investigate MCP-1 production in culture medium, TMJSCs were stimulated with 0.01 ng/ml IL-1β for 27–28 h (Figure 2B). Especially in Figure 4B, in order to investigate MCP-1 or IL-1β production in culture medium, TMJSCs and RAW264.7 cells were cultured as described in ‘TMJSC and RAW264.7 cell co-culture’ section below. After the culture, cells were centrifuged at 14600 rpm for 10 min, the supernatants were collected and stored at –80°C until subsequent measurement. Levels of MCP-1 and IL-1β protein were measured using Quantikine® ELISA kits for Mouse/Rat CCL2/JE/MCP-1 and Mouse IL-1β/IL-1F2 (R&D systems, Minneapolis, MN, U.S.A.) according to the manufacturer’s instructions. Absorbance was recorded at 450 nm (570 nm as a reference wavelength) on an iMark microplate reader (Bio-Rad, CA, U.S.A.).

### Cell adhesion assay

TMJSCs (5.3 × 10^3^) were seeded on a 12-mm round coverslip (Matsunami, Osaka, Japan) in 24-well plates and incubated in 0.5-ml DMEM containing 20% FBS and rhFGF-2 (10 ng/ml) for 3 days. Then, after the cells were serum-starved (cultured in DMEM without FBS) and incubated overnight (12–17 h), they were stimulated with 0.1 ng/ml IL-1β in DMEM without FBS for 24 h. Then, RAW264.7 cells (1 × 10^5^ cells/well) were layered on to IL-1β-stimulated TMJSCs monolayers and incubated for 30 min in a humidified atmosphere of 5% CO_2_ at 37°C to allow RAW264.7 cell–TMJSC adhesion. Next, the non-adhesive RAW264.7 cells were removed by twice washing co-culture wells with culture medium. Then, the adherent RAW264.7 cells and TMJSCs were fixed and immunostained with anti-CD45 and anti-GFP antibodies, respectively, and then both these cell types were stained with DAPI. Finally, the cells that had adhered to the TMJSC monolayer were observed and microphotographed using a laser confocal microscope (C1 si, Nikon, Tokyo, Japan). The adhesive RAW264.7 cells, which were immunostained by anti-CD45, were counted on the images of the microphotographs.

### Immunofluorescence

The cells were fixed in methanol/acetone (1:1) for 3 min at −20°C and subsequently washed three times with PBS. Blocking of non-specific antibody binding to the fixed cells was performed with 1% BSA (Sigma–Aldrich, St. Louis, MO, U.S.A.) in PBS overnight at 4°C. The fixed samples were incubated with the primary antibodies described above for 1 h at room temperature. Then, they were washed three times with PBS, followed by incubation for 30 min with the appropriate fluorescent secondary antibodies at room temperature. For the experiment in Figure 3A, they were incubated with Alexa Fluor® 488 phalloidin for another 30 min. Samples were viewed and photos taken on a laser confocal microscope (C1 si, Nikon, Tokyo, Japan).

### TMJSC and RAW264.7 cell co-culture

In Figure 4B, in order to elucidate existence of a positive amplifying loop between IL-1β-treated TMJSCs and RAW264.7 cells, we evaluated the protein concentration of MCP-1 in the conditioned medium from monocultures of RAW264.7 cells and IL-1β-treated TMJSCs, and evaluated that in the conditioned medium from co-cultures between IL-1β-treated TMJSCs and RAW264.7 cells with or without cell–cell contact. We also evaluated the protein concentrations of IL-1β in the conditioned medium from these monocultures and co-cultures.

For preparation of the conditioned medium from IL-1β-treated TMJSCs monoculture, TMJSCs were seeded in 24-well cell culture insert companion plates and stimulated with IL-1β, as described in the ‘IL-1β stimulation of TMJSCs’ section. Then, the IL-1β-stimulated TMJSCs were washed by DMEM once for the removal of excess amount of IL-1β. And then the fresh DMEM without IL-1β was added to each well. Then, the transwell devices (0.4 μm pore size) were inserted into the individual wells of the plates. And then, DMEM was added on to the top of the membranes without seeding any RAW264.7 cells on to the top of the membranes. Then, the IL-1β-removed TMJSC monoculture was maintained further for 24 h for the collection of the conditioned medium. On the other hand, for preparation of the conditioned medium from RAW264.7 cells monoculture, DMEM supplemented with 0.1 ng/ml of IL-1β was added into each well in 24-well cell culture insert companion plates without seeding of any TMJSCs, and subsequently maintained for 24 h. After the washing of each well by DMEM once for the removal of IL-1β, the fresh DMEM without IL-1β was added to each well. And then, the transwell devices were inserted into the individual wells of the plates. RAW264.7 cells (1 × 10^5^ cells/well) were seeded on the top of the membranes and cultured further for 24 h.

For the preparation of conditioned medium from the co-culture between IL-1β-treated TMJSCs and RAW264.7 cells without cell–cell contact, TMJSCs were seeded in 24-well cell culture insert companion plates and stimulated with IL-1β, as described in the ‘IL-1β stimulation of TMJSCs’ section. Then, the IL-1β-stimulated TMJSCs were washed by DMEM once for the removal of excess amount of IL-1β. And then, the fresh DMEM without IL-1β was added to each well. Then, the transwell devices were inserted into the individual wells of the plates. RAW264.7 cells (1 × 10^5^ cells/well) were seeded on the top of the membranes and cultured further for 24 h. On the other hand, for the preparation of conditioned medium from the co-culture between IL-1β-treated TMJSCs and RAW264.7 cells with cell–cell contact, TMJSCs were seeded in 24-well cell culture insert companion plates and stimulated with IL-1β, as described in the ‘IL-1β stimulation of TMJSCs’ section. Then, the IL-1β-stimulated TMJSCs were washed with DMEM once for the removal of excess amount of IL-1β. And then, RAW264.7 cells (1 × 10^5^ cells/well) were seeded and layered on to the IL-1β-removed TMJSCs monolayer and co-cultured for an additional 24 h.

### Statistical analysis

Data were presented as means ± S.D. (*n*=4 for Figures 2A,B, 3C, and 4B; *n*=8 for control, and *n*=7 for rmMCP-1 in Figure 2D; *n*=8 for Figure 3B; and *n*=10 for Figure 2E). Statistical significance was analyzed by Student’s *t* test (Figure 3B), Welch’s *t* test (Figure 2B,D), or Tukey’s multiple comparison test (Figures 2A,E, 3C, and 4B) (GraphPad Prism Software ver.7, San Diego, CA). Values of *P<0.05, **P<0.01, ***P<0.001, and ****P<0.0001 were considered to be statistically significant. The results shown in all experiments were representative of at least two independent experiments.

## Results

### Confirmation of fibroblastic character of TMJSCs

**Figure 1:**
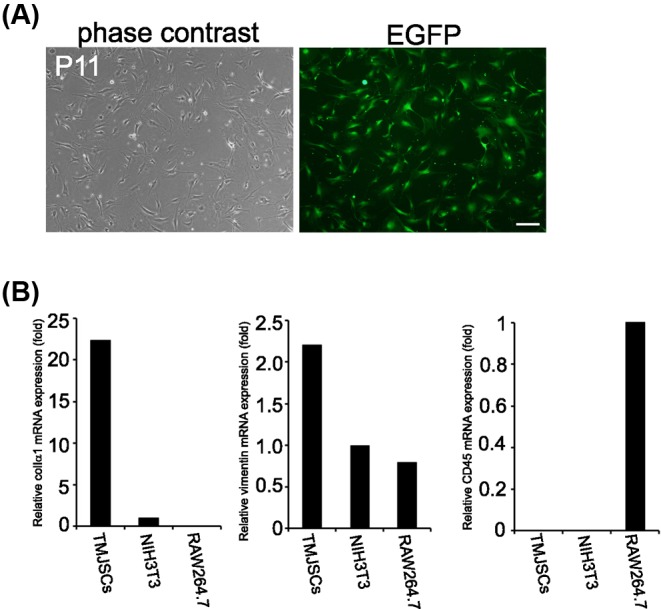
TMJSCs exhibit fibroblastic character but do not exhibit leukocyte character (**A**) Morphology of cultured TMJSCs obtained from a 10-week-old EGFP mouse at passage 11 (phase contrast, left picture; EGFP, right picture). After the soft tissues were collected from around male EGFP mouse TMJ, TMJSCs were obtained by the outgrowth method, as described in ‘Materials and methods’ section. TMJSCs were cultured and passaged in 20% FBS in DMEM supplemented with rhFGF-2 (10 ng/ml). Scale bar, 200 μm. (**B**) Characterization of TMJSCs. TMJSCs, NIH3T3 cells, and RAW264.7 cells were cultured and grown to 80% confluence, then total RNA was isolated from each culture. mRNA expression patterns of colIα1 and vimentin (mesenchymal cells markers) or CD45 (leukocyte common antigen) in cultured TMJSCs, NIH3T3 cells, and RAW264.7 cells were evaluated by RT-qPCR. The mRNA expression levels of these genes were normalized to those of 18S rRNA, and the relative expression levels are shown as fold-increases or decreases relative to the level in NIH3T3 cells (colIα1 and vimentin) or RAW264.7 cells (CD45).

To elucidate the relationship between TMJ tissue cells and inflammatory cells, we obtained TMJSCs from EGFP mouse TMJ tissues. Morphologically, the cultured TMJSCs appeared to be spindle-shaped fibroblasts when observed by phase-contrast microscopy, and all of them were EGFP positive (Figure 1A). Moreover, we confirmed the fibroblastic character of TMJSCs by quantitative reverse transcriptase-PCR (RT-qPCR) analysis. TMJSCs highly expressed mRNA of *colIα1* and *vimentin* compared with NIH3T3 cells as a standard fibroblast control, and hardly expressed that of *CD45*, a leukocyte common antigen, whereas RAW264.7 cells, a murine monocyte/macrophage cell line expressed that of *CD45* (Figure 1B). These results suggested that TMJSCs had mesenchymal and fibrogenic phenotypes.

### MCP-1 secreted from IL-1β-stimulated TMJSCs promotes migratory activity of RAW264.7 cells

**Figure 2:**
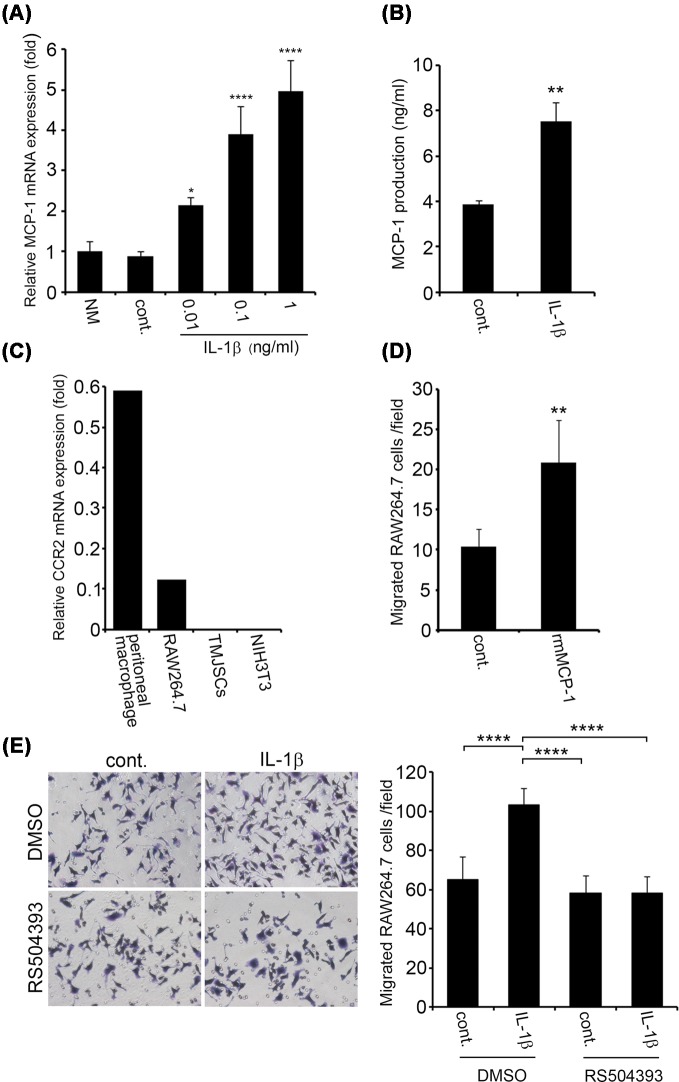
MCP-1 secreted from IL-1β-stimulated TMJSCs promotes migratory activity of RAW264.7 cells (**A**) *Dose-dependent response of MCP-1 mRNA expression by TMJSCs to IL-1β stimulation.* TMJSCs were stimulated for 4 h with 0.01–1 ng/ml of IL-1β. The mRNA expression levels of *MCP-1* were normalized to those of *GAPDH* and the relative expression levels are shown as fold-increases or decreases relative to the level in controls. (**B**) *MCP-1 production from IL-1β-stimulated TMJSCs.* TMJSCs were stimulated for 27 h with 0.01 ng/ml of IL-1β, and the conditioned medium was collected. The protein level of MCP-1 in the conditioned medium was measured by ELISA, as described in the ‘Materials and methods’ section. The relative production levels are shown as fold-increases or decreases relative to the level in controls. (A,B) Each value represents the mean ± S.D. (*n*=4). Similar results were obtained in two independent experiments. *P<0.05, **P<0.01, and ****P<0.0001 were considered significant compared with the control. (**C**) *CCR2 mRNA expression in RAW264.7 cells and primary macrophages.* The mRNA expression levels of *CCR2* in C57BL/6J mouse splenocytes, resident peritoneal macrophages, RAW264.7 cells, TMJSCs, and NIH3T3 cells were evaluated by RT-qPCR. The levels were normalized to those of 18S rRNA and the relative expression levels are shown as fold-increases or decreases relative to the levels in C57BL/6J mouse splenocytes (data from C57BL/6J mouse splenocytes as a control were not shown). C57BL/6J mouse splenocytes and NIH3T3 cells were used as positive or negative controls, respectively. (**D**) *Chemotaxis assay of RAW264.7 cells in response to rmMCP-1, as described in ‘Materials and methods’ section.* Seven or eight different fields with migrated cells were recorded as photomicrographs, and the migrated cell numbers were counted for each field (*n*=8 for control; *n*=7 for rmMCP-1). Each value represents the mean ± S.D. Similar results were obtained in two independent experiments. **P<0.01 was considered significant compared with the control. (**E**) *Effects of CCR2 selective antagonist, RS504393 on TMJSCs-secreted chemotactic factor-induced migration of RAW264.7 cells.* This assay was carried out using transwell cell culture inserts, as described in ‘Materials and methods’ section. Ten different fields with migrated cells were recorded as photomicrographs, and the migrated cell numbers were counted for each field (*n*=10). Each value represents the mean ± S.D. Values of ****P<0.0001 were considered to be statistically significant.

Next, we examined whether TMJSCs express and produce homing factors, which possibly induce chemotactic activities and mobilization of inflammatory cells into the damaged TMJ sites. Recently, it has been reported that the production of MCP-1 is increased in human TMJ synoviocytes stimulated with IL-1β [8]. MCP-1 is one of the representative chemokines that induce monocyte/macrophage migration into inflammation sites [11]. Therefore, we stimulated TMJSCs with IL-1β and then evaluated the status of MCP-1 synthesis in these TMJSCs. The *MCP-1* mRNA levels were increased in response to IL-1β (0.01–1 ng/ml) in a dose-dependent manner 4 h after stimulation (Figure 2A). Moreover, the protein level of MCP-1 in conditioned medium from TMJSCs stimulated with 0.01 ng/ml IL-1β was determined by ELISA (Figure 2B). The IL-1β-stimulated TMJSCs secreted greater amounts of MCP-1 than the unstimulated controls 27 h after stimulation. Thus, these results indicated that IL-1β similarly promoted MCP-1 expression in TMJSCs derived from mice, as previously demonstrated for human TMJSCs [8]*.* MCP-1 binds to the cell surface CCR2 [12–14] and has important roles in monocyte recruitment to inflammation sites [15]. It has been reported that CCR2 is expressed in monocytes, T cells, dendritic cells, fibroblasts, and endothelial cells [16]. By RT-qPCR analysis, RAW264.7 cells were confirmed to express *CCR2* mRNA (Figure 2C), whereas mouse peritoneal macrophages expressed larger amount of *CCR2* mRNA than RAW264.7 cells by 4.8-times. We examined chemotactic activity of RAW264.7 cells stimulated with rmMCP-1. As shown in Figure 2D, rmMCP-1 (100 ng/ml) significantly enhanced RAW264.7 cell migration. We examined whether RAW264.7 cells migrated in response to factors secreted from the IL-1β-stimulated TMJSCs. The number of cells that migrated into the lower chamber in which the IL-1β-stimulated TMJSCs were cultured was greater than that of cells that migrated into the lower chamber in which unstimulated TMJSCs were cultured (Figure 2E). Intriguingly, CCR2 selective antagonist RS504393 significantly abrogated the IL-1β-stimulated TMJSCs-promoted migration of RAW264.7 cells, indicating tha**t** MCP-1 secreted from IL-1β-stimulated TMJSCs promotes migratory activity of RAW264.7 cells.

### Inflammatory stimulation against TMJSCs promotes adhesion of RAW264.7 cells to TMJSCs possibly through augmentation of ICAM-1/LFA-1 (CD11a/CD18)-mediated cell–cell adhesion

**Figure 3:**
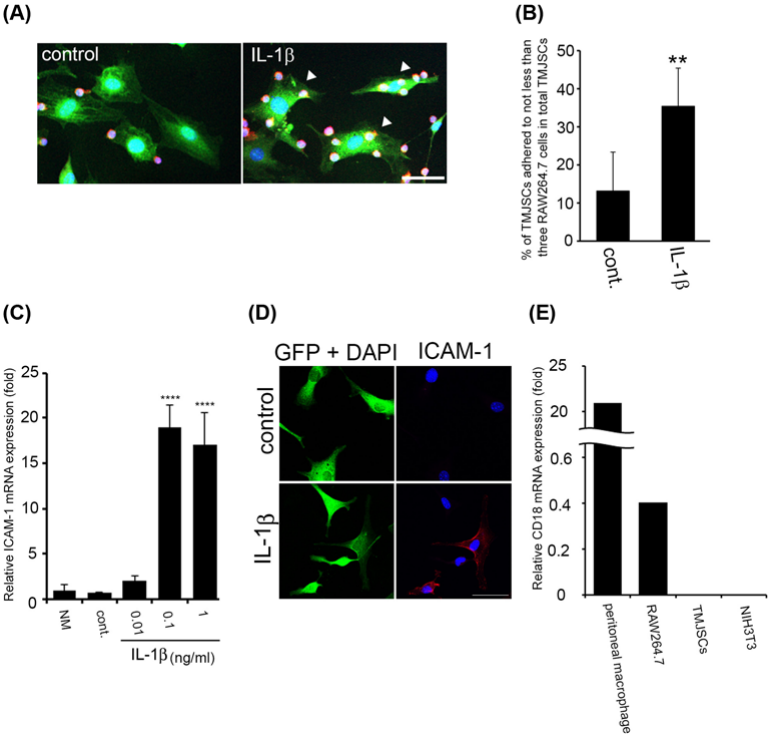
IL-1β promotes adhesion of TMJSCs to RAW264.7 cells (**A**) *RAW264.7 cell adhesion assay for IL-1β-stimulated TMJSCs.* RAW264.7 cells were layered over IL-1β-stimulated TMJSCs. Unbound cells were removed and immunostained with anti-CD45, anti-GFP, phalloidin, and DAPI (red, anti-CD45; green, anti-GFP or phalloidin; blue, DAPI). Scale bar, 50 μm. (**B**) *Effect of IL-1β on the ability of TMJSCs to adhere to RAW264.7 cells.* TMJSCs adhered to not less than three CD45-positive cells, which localized on GFP and phalloidin-positive areas, was counted in eight different fields. Each value represents the mean ± S.D. (*n*=8). Similar results were obtained in two independent experiments. **P<0.01 was considered significant compared with the control. (**C**) *Dose-dependent response of mRNA expression of ICAM-1 in TMJSCs to IL-1β stimulation.* TMJSCs were seeded in each well of 24-well plates and stimulated for 4 h with 0.01–1 ng/ml of IL-1β. The mRNA expression level of *ICAM-1* was normalized to that of *GAPDH*. Each value represents the mean ± S.D. (*n*=4). ****P<0.0001 was considered significant compared with the control. (**D**) *Immunostaining of ICAM-1 in TMJSCs stimulated with IL-1β.* TMJSCs were seeded on 12-mm round coverslips in 24-well plates for 3 days. Then, after TMJSCs were starved, the cells were stimulated with 0.1 ng/ml IL-1β for 24 h. The cells were then fixed in methanol/acetone and immunostained with anti-ICAM-1 and anti-GFP (red, anti-ICAM-1; green, anti-GFP; and blue, DAPI). Scale bar, 50 μm. (**E**) *CD18 mRNA expression in RAW264.7 cells and primary macrophages.* The mRNA expression levels of *CD18* in C57BL/6J mouse splenocytes, resident peritoneal macrophages, RAW264.7 cells, and NIH3T3 cells were evaluated by RT-qPCR. The levels were normalized to those of 18S rRNA and the relative expression levels are shown as increases or decreases relative to the level in C57BL/6J mouse splenocytes (data from C57BL/6J mouse splenocytes as a control were not shown). C57BL/6J mouse splenocytes and NIH3T3 cells were used as positive or negative controls, respectively.

To investigate cell–cell interactions between TMJSCs and monocytes/macrophages in inflamed TMJ tissue, we examined the adhesive status of RAW264.7 cells to TMJSCs cultured with or without IL-1β stimulation. Intriguingly, the number of RAW264.7 cells that adhered to IL-1β-stimulated TMJSCs was greater than that of cells adhered to unstimulated TMJSCs (Figure 3A,B). In addition, we investigated how RAW264.7 cells preferentially adhere to IL-1β-stimulated TMJSCs at the molecular level. Generally, it has been reported that leukocyte–mesenchymal cell adhesion is mediated via α- and β-integrins on leukocytes and adhesion molecules such as ICAMs on mesenchymal cells [17]. Therefore, we investigated the expression status of these adhesion molecules in the IL-1β-stimulated TMJSCs. IL-1β increased mRNA expression of *ICAM-1* in TMJSCs 4 h after stimulation (0.1–1 ng/ml) (Figure 3C). In addition, immunofluorescence analysis revealed that IL-1β (0.1 ng/ml) increased ICAM-1 expression at the protein level 24 h after stimulation (Figure 3D). It has been reported that ICAM-1 binds to leukocyte function-associated antigen (LFA)-1 (CD11a/CD18) that is known to be an important integrin as a leukocyte adhesion molecule [18]. Then, we confirmed that *CD18* (*integrin β2*) mRNA expression in RAW264.7 cells and primary macrophages by RT-qPCR analysis (Figure 3E). We found that mouse peritoneal macrophages expressed larger amount of *CD18* mRNA than RAW264.7 cells by 51.7-times.

### Cell–cell interactions mediated through soluble factors and cell–cell adhesion molecules between IL-1β-stimulated TMJSCs and RAW264.7 cells synergistically augment secretion of MCP-1 from these cells

**Figure 4:**
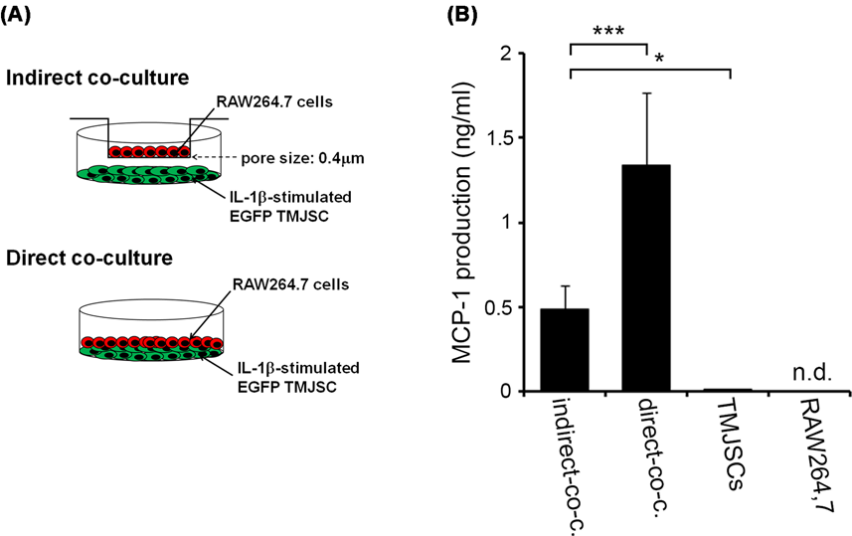
Cell–cell interactions mediated through soluble factors and cell–cell adhesion molecules between IL-1β-stimulated TMJSCs and RAW264.7 cells synergistically augment secretion of MCP-1 from these cells (**A**) Schema of the direct or indirect co-culture system. (**B**) MCP-1 production in conditioned medium from monocultures and direct or indirect co-cultures of IL-1β-stimulated TMJSCs and RAW264.7 cells. For preparation of the conditioned medium from IL-1β-treated TMJSC monoculture, TMJSCs were first stimulated for 24 h with 0.1 ng/ml of IL-1β. The IL-1β-stimulated TMJSCs were subsequently washed for the removal of IL-1β. Then, the fresh medium without IL-1β was added to each well to obtain conditioned medium. Then, the transwell devices were inserted into the individual wells of the plates. And then, the fresh medium without IL-1β was added on to the top of the membranes without seeding any RAW264.7 cells on to the top of the membranes. Then, the IL-1β-removed TMJSCs monoculture was maintained further for 24 h. On the other hand, for preparation of the conditioned medium from RAW264.7 cells monoculture, fresh medium with 0.1 ng/ml of IL-1β was added into each well in 24-well cell culture insert companion plates without seeding of any TMJSCs, and subsequently maintained for 24 h. After the washing of each well of the culture plates, the fresh medium without IL-1β was added to the each well. Then, the transwell devices were inserted into the individual wells of the plates. RAW264.7 cells (1 × 10^5^ cells/well) were subsequently seeded on the top of the membranes and cultured further for 24 h. For the preparation of conditioned medium from the co-culture between TMJSCs and RAW264.7 cells without cell–cell contact, TMJSCs were first stimulated for 24 h with 0.1 ng/ml of IL-1β. The IL-1β-stimulated TMJSCs were subsequently washed for the removal of IL-1β. Then, the fresh medium without IL-1β was added to each well. Then, the transwell devices were inserted into the individual wells of the plates. RAW264.7 cells (1 × 10^5^ cells/well) were seeded on the top of the membranes and cultured further for 24 h. On the other hand, for the preparation of conditioned medium from the co-culture between IL-1β-treated TMJSCs and RAW264.7 cells with cell-cell contact, TMJSCs were first stimulated for 24 h with 0.1 ng/ml of IL-1β. The IL-1β-stimulated TMJSCs were subsequently washed for the removal of IL-1β. Then, RAW264.7 cells (1 × 10^5^ cells/well) were seeded and layered on to the IL-1β-stimulated TMJSCs monolayer and co-cultured for an additional 24 h. Each value represents the mean ± S.D. (*n*=4). *P<0.05 and ***P<0.001 were considered statistically significant.

Previous study has shown that MCP-1 is produced by cell–cell contact between human lung fibroblasts and monocytes from peripheral blood [19]. Therefore, we investigated whether MCP-1 synthesis was promoted by cell–cell contact between TMJSCs and RAW264.7 cells. We compared the amounts of secreted MCP-1 in the conditioned medium from co-cultured TMJSCs and RAW264.7 cells with or without cell–cell contact (Figure 4A). To avoid direct cell–cell contact between TMJSCs and RAW264.7 cells as a control experiment, we carried out the co-culture assay using a 0.4-μm pore-size cell culture insert system described in the ‘Materials and methods’ section.

In order to examine whether there was amplifying loop mediated through soluble factors or adhesion molecules between macrophage lineage cells and TMJSCs, we evaluated the protein concentrations of MCP-1 in the conditioned media from monocultures of IL-1β-treated TMJSCs and RAW264.7 cells, and also evaluated those in the conditioned media from co-cultures between IL-1β-treated TMJSCs and RAW264.7 cells with or without cell–cell contact as described in ‘Materials and methods’ section. We found that the concentration of MCP-1 protein in the conditioned medium from monoculture of IL-1β-treated TMJSCs was at 10.5 pg/ml, whereas that of non-treated RAW264.7 cells was not at detectable level (Figure 4B). Intriguingly, production of MCP-1 was dramatically increased by co-culture between IL-1β-treated TMJSC and RAW264.7 cells without cell–cell contact compared with that by monoculture of IL-1β-treated TMJSC. Moreover, production of MCP-1 was clearly increased by co-culture between IL-1β-treated TMJSC and RAW264.7 cells with cell–cell contact compared with that by co-culture without cell–cell contact (Figure 4B). In addition, ELISA analysis revealed that the concentrations of IL-1β protein in the conditioned media from monocultures of IL-1β-treated TMJSCs and non-treated RAW264.7 cells and the concentrations of that in the conditioned media from co-cultures between IL-1β-treated TMJSC and non-treated RAW264.7 cells with or without cell–cell contact were not at detectable level (data not shown).

## Discussion

A previous study has implicated that CD45RO- or CD68-positive cells infiltrate into the synovium of patients with painful joint clicking or OA; in particular, CD68-positive cells are more abundant in OA patients [20]. It is generally known that CD68 is a representative antigen for macrophage detection [21]. The infiltration of monocytes/macrophages to diseased tissues is a characteristic finding in chronic inflammation, and it is thought that TMJ synoviocytes and monocytes/macrophages may have some kind of communication with each other at such sites. Therefore, to clarify the relationship between TMJ synoviocytes and monocytes/macrophages in TMJ inflammation, we obtained and cultured TMJSCs from murine TMJ areas (Figure 1A,B). Several research groups have reported how to isolate TMJ synoviocytes from TMJ synovial membranes, and the isolated cells exhibit spindle-like shapes and fibroblast-like or mesenchymal stem cell-like characteristics [22–24]. Anatomically, it is reported that there are two types of TMJ synovial cells: macrophage-like cells (type A) and fibroblast-like cells (type B) [2,25]. Tobe et al. [22] reported that primary cultured synovial cells obtained from human TMJ tissue were positively immunostained with certain fibroblastic marker antibodies (propyl 4-hydroxylase and vimentin), whereas macrophage markers were negative for these stains. TMJSCs isolated from mouse TMJ tissues also expressed fibroblastic markers, collagen type I and vimentin, but not the leukocyte marker CD45 (Figure 1B). Therefore, it was suggested that TMJSCs might be type B cells (fibroblast-like cells). To investigate cell–cell interactions between TMJSCs and monocytes/macrophages, we used murine macrophage cell line RAW264.7 cells instead of primary cultures of mouse monocytes/macrophages expected to home to TMJ inflammation sites *in vivo*. Recently, it has been reported that MCP-1 production is increased when fibroblast-like synoviocytes obtained from patients with TMD are stimulated with IL-1β [8]. In accordance with this report, we also confirmed that MCP-1 production by mouse TMJSCs was facilitated by IL-1β stimulation (Figure 2A,B). We also examined that RAW264.7 cells express CCR2, the receptor for MCP-1 (Figure 2C). In addition, rmMCP-1 actually promoted migratory activity of RAW264.7 cells (Figure 2D). Moreover, CCR2 selective antagonist RS504393 suppressed the IL-1β-stimulated TMJSCs-promoted migration of RAW264.7 cells, indicating tha**t** MCP-1 secreted from IL-1β-stimulated TMJSCs promotes migratory activity of RAW264.7 cells (Figure 2E). Therefore, it was strongly suggested that TMJSCs stimulated with the inflammatory cytokine IL-1β possibly recruited monocytes/macrophages to inflamed synovial membranes through secretion of MCP-1 at TMJ inflammation sites.

It is generally known that immune cells such as macrophages, T cells, and mast cells infiltrate inflamed synovial tissue in OA [26]. The first step of the infiltration of immune cells into synovial tissue is adhesion of immune cells to synovial membranes. Therefore, we examined how IL-1β stimulation of TMJSCs affected cell–cell adhesion between monocytes/macrophages and TMJSCs. A cell adhesion assay revealed that the number of TMJSCs adhered to RAW264.7 cells was increased after TMJSCs were stimulated with IL-1β (Figure 3A,B). Actually, IL-1β increased ICAM-1 expression in TMJSCs possibly bound to LFA-1 on RAW264.7 cells (Figure 3C–E). However, it was not possible to rule out the possibility that IL-1β directly stimulated RAW264.7 cells, resulting in augmentation of adhesive ability in RAW264.7 cells to TMJSCs. Furthermore, we investigated whether the cell–cell interaction between TMJSCs and monocytes/macrophages affected the status of progression of inflammation in synovial tissue. We found that production of MCP-1 was dramatically increased by co-culture between IL-1β-treated TMJSC and RAW264.7 cells without cell–cell contact compared with that by monocultures of IL-1β-treated TMJSC or RAW264.7 cells (Figure 4B). Moreover, production of MCP-1 was clearly increased by co-culture between IL-1β-treated TMJSC and RAW264.7 cells with cell–cell contact compared with that by the co-culture without cell–cell contact (Figure 4B). In addition, ELISA analysis revealed that the concentrations of IL-1β protein in the conditioned media from monocultures of IL-1β-treated TMJSCs and non-treated RAW264.7 cells, and the concentrations of that from co-cultures between IL-1β-treated TMJSC and non-treated RAW264.7 cells with or without cell–cell contact were not at detectable level (data not shown). These results strongly suggested that cell–cell interactions mediated through soluble factors other than IL-1β and cell–cell adhesion molecules between IL-1β-stimulated TMJSCs and RAW264.7 cells synergistically augments secretion of MCP-1 from these cells: (i) there was a positive amplifying loop for MCP-1 production between inflamed TMJSCs and macrophage lineage cells mediated through soluble factors other than IL-1β, and that (ii) cell–cell contact between inflamed TMJSCs and macrophage lineage cells further up-regulated MCP-1 production in the inflammatory sites in the TMJ. Previous studies have reported that interactions of lung fibroblasts and monocytes from peripheral blood [19] or renal fibroblasts and peripheral blood mononuclear cells [27] facilitate the production of MCP-1.

We also found that mouse peritoneal macrophages expressed larger amount of CCR2 (Figure 2C) and CD18 (Figure 3E) than RAW264.7 cells, which was not inconsistent with our hypothesis that cell–cell interactions between monocytes/macrophages and synoviocyte-like cells through ICAM-1/LFA-1 (CD11a/CD18) promote inflammatory cell infiltration mediated by augmentation of MCP-1 production in TMJ.

Taken together, it was suggested that TMJSCs stimulated with inflammatory cytokine IL-1β in inflamed synovial membranes vigorously secreted MCP-1, which exerted further homing of monocytes/macrophages into inflamed TMJ synovial tissue, resulting in the elevated adhesion of monocytes/macrophages to the synovial membrane. IL-1β up-regulated the expression level of ICAM-1 on TMJSCs bound to LFA-1 (CD11a/CD18) on monocytes/macrophages in inflamed synovial membranes, suggesting that IL-1β up-regulated the adhesive ability of TMJSCs to monocytes/macrophages. Thus, the cell–cell interactions between inflammatory cells and TMJSCs, which was mediated by a soluble factor such as MCP-1 or adhesive molecules such as ICAM-1 and LFA-1 on the surface of TMJSCs and inflammatory cells, respectively, possibly exacerbates the symptoms of synovitis or promotes its transition to chronic inflammation in TMJ. Our findings partially clarify the molecular mechanisms underlying the progression of inflammation in TMJ, and may aid in identifying drug targets for treating this condition at the molecular level.

## Abbreviations

CCR2: CC chemokine receptor 2
colIα1: α1 chain of collagen type I
DMEM: Dulbecco’s modified Eagle’s medium
ICAM-1: intracellular adhesion molecule 1
IL-1β: interleukin 1β
LFA-1: leukocyte function-associated antigen-1
MCP-1: monocyte chemoattractant protein 1
OA: osteoarthritis
rhFGF-2: recombinant human fibroblast growth factor 2
rmMCP-1: recombinant mouse MCP-1
RT-qPCR: quantitative reverse transcriptase-PCR
TMD: temporomandibular disorder
TMJ: temporomandibular joint
TMJSC: TMJ synoviocyte-like cell
